# Toxicity of the Covalent Organic Framework Material TpPa to Zebrafish

**DOI:** 10.3390/ani16152282

**Published:** 2026-07-23

**Authors:** Cuiming Huang, Jingui Lin, Xiudan Yang, Lu Gan, Lijie Xu, Muting Yan, Han Gong

**Affiliations:** 1 Joint Laboratory of Guangdong Province and Hong Kong Region on Marine Bioresource Conservation and Exploitation, South China Agricultural University, Guangzhou 510642, China; huangcuiming0124@163.com (C.H.); jingui_lin@163.com (J.L.); yangxiudan0020@163.com (X.Y.); 2 College of Materials Science and Engineering, Nanjing Forestry University, Nanjing 210037, China; ganlu@njfu.edu.cn; 3 College of Ecology and Environment, Nanjing Forestry University, Nanjing 210037, China; xulijie@njfu.edu.cn; 4 Department of Chemistry, Stockholm University, 10691 Stockholm, Sweden

**Keywords:** covalent organic framework, zebrafish embryo, *Danio rerio*, safety assessment, toxicity

## Abstract

Covalent organic frameworks (COFs) show great potential in environmental remediation, yet the aquatic safety of TpPa (Tp = 1,3,5-triformylphloroglucinol, Pa = *p*-phenylenediamine), a typical COF, remains unclear. This study employed zebrafish (*Danio rerio*) to evaluate the environmental toxicity of TpPa and elucidate molecular mechanisms. Zebrafish embryos were exposed to 0–50 mg/L TpPa for 120 h, and developmental toxicity, oxidative stress, and physiological and molecular responses were investigated. Results demonstrated that TpPa exposure exerted concentration- and time-dependent effects. Mortality and malformation rates increased significantly from 5 mg/L, accompanied by reduced body length. The hatching rate showed a biphasic pattern, increasing with concentration at 48 h and decreasing with concentration at 120 h. Spontaneous movement was elevated at 50 mg/L, while heart rate increased dose-dependently starting from 5 mg/L at 48 h and from 1 mg/L at 72 h. Mechanistically, TpPa induced oxidative stress via reactive oxygen species accumulation and impaired the activities of both superoxide dismutase and catalase. These effects activated the *p53*-mediated apoptotic pathway, reduced the *Bcl2*/*Bax* ratio, and disrupted the *GH*/*IGF* growth axis and neuromotor regulatory pathways, ultimately causing developmental abnormalities. These findings provide critical data for the environmental safety assessment of TpPa, with direct relevance to establishing health monitoring protocols for COF-exposed aquaculture species.

## Simple Summary

Covalent organic frameworks (COFs) are promising materials for environmental cleanup due to their unique structures. However, little is known about their safety for aquatic life once they are released into water. This study used zebrafish (*Danio rerio*), a small freshwater fish commonly used in toxicity testing, to evaluate the harmful effects of a typical COF material called TpPa (Tp = 1,3,5-triformylphloroglucinol, Pa = *p*-phenylenediamine). Zebrafish embryos and larvae were exposed to different concentrations of TpPa for 120 h. The results showed that TpPa caused damage in a dose-dependent manner, with obvious developmental effects observed, including delayed hatching, physical deformities, reduced growth, abnormal heart rates, and altered swimming behavior. Further investigation revealed that TpPa induced oxidative stress and activated cell death pathways, ultimately disrupting normal development through a cascade mechanism. These findings provide important information for setting safety limits and assessing the ecological risks of TpPa and similar COF materials in aquatic environments.

## 1. Introduction

Covalent organic frameworks (COFs) are a class of ordered porous crystalline materials composed of light elements such as C, H, and N linked by reversible covalent bonds, featuring a large specific surface area, low density, tunable pore size and excellent structural stability [1]. Nanomaterials are defined as materials with at least one external dimension in the 1–100 nm range or internal structure at the nanoscale [2]. Given that COFs typically possess nanoscale pore dimensions (commonly 2–50 nm) and can be synthesized as nanosheets with thicknesses falling within the nanoscale range, they are generally considered as a subclass of nanomaterials [3]. Since the first report by Côté and colleagues in 2005, COFs have emerged as a premier class of crystalline porous materials [4]. Over the past two decades, the field has witnessed exponential growth, with thousands of distinct frameworks reported and tens of thousands of research articles published [5]. Owing to their structural modularity and permanent porosity, COFs have been extensively applied in catalysis, energy storage, environmental remediation, and so on [6].

Based on different linkage modes, COFs are mainly classified into four categories, including boron-containing, imine-based, triazine-based, and polyimide-based COFs [7]. As a typical representative of imine-linked COFs, TpPa (Tp = 1,3,5-triformylphloroglucinol, Pa = *p*-phenylenediamine) is a β-ketoenamine-linked COF synthesized via the Schiff base condensation reaction of 1,3,5-triformylphloroglucinol with *p*-phenylenediamine or 2,5-dimethyl-*p*-phenylenediamine [8]. Its unique enol-to-keto tautomerism endows TpPa with exceptional chemical stability, enabling structural integrity under harsh conditions, including strong acids, strong alkalis, and boiling water [9]. To date, hundreds of studies have reported on the synthesis, modification, and application of TpPa-based materials, including pristine TpPa-1 and TpPa-2, as well as their composites with metals, magnetic nanoparticles, and other COFs [10]. These materials have demonstrated broad applicability in key areas such as membrane separation, adsorption, photocatalysis, energy storage, and sensing [11,12,13]. For instance, TpPa exhibits a maximum adsorption capacity of approximately 310.8 mg/g for Cr(VI) with stable reusability [14]. Moreover, sulfonated TpPa (TpPa-SO_3_Na) achieves rapid removal of norfloxacin (~99%), ciprofloxacin (~98%), tetracycline (~91%), and methylene blue (~96%) within 1 min [15].

However, the aforementioned environmental application studies have primarily focused on the functional development and performance optimization of COF materials, with relatively insufficient attention to their potential ecological risks upon environmental release. Currently, toxicological investigations of COFs have predominantly concentrated on in vitro cytotoxicity in human cells, mainly within biomedical applications such as drug delivery, bioimaging, and photothermal therapy [11,16,17,18,19,20,21]. Most studies have suggested that COFs exhibit low cytotoxicity to normal or tumor cells, causing no significant cell damage even at a high concentration of 100 μg/mL [22,23]. In vivo experiments in mice have also confirmed no significant toxic reactions at certain doses, demonstrating favorable biocompatibility [24].

Notably, these safety assessments in the biomedical field cannot be directly extrapolated to environmental exposure scenarios [25]. As a representative COF widely applied in environmental remediation, TpPa is typically synthesized as insoluble powders that are either directly dispersed in aqueous systems for adsorption or processed into membranes and nanosheets for water treatment [26,27,28,29]. Such particulate materials are difficult to separate and recycle during use and may inevitably be released into natural environments such as soil and water, resulting in environmental contamination that can negatively impact both individual organisms and the food chain [10,30]. In fact, environmental toxicity research in the COFs field is extremely scarce. Only a limited number of studies have investigated the phenotypic toxicity of TpBD-Me_2_ COF to zebrafish (*Danio rerio*) embryos, revealing that toxicity primarily occurs post-hatching, with exposure to a low concentration (0.001 μg/mL) potentially posing moderate environmental risks through blockage of embryonic chorionic pores [23]. Therefore, systematic evaluation of the toxicological effects of TpPa on model organisms is not only an urgent need to fill the gap in environmental safety assessment of this material, but also an important entry point for improving the ecological risk assessment system of COF substances.

Zebrafish is an ideal model organism in ecotoxicological research, characterized by small size, high fecundity, embryonic transparency, and external fertilization and development, enabling multidimensional toxicity assessment of pollutants at phenotypic, physiological, biochemical, and molecular levels [31]. Numerous studies have documented the toxic effects on embryonic development and the underlying mechanisms of various nanomaterials differing in size and composition [32,33]. As a representative vertebrate model, zebrafish provides critical insights into developmental toxicity and organ-specific damage, offering essential evidence for assessing the potential risks of TpPa to higher aquatic organisms.

This study aims to fill the gap in toxicological data regarding the effects of COFs on aquatic organisms and employs zebrafish as the model organism to systematically evaluate the potential risks of TpPa in aquaculture water environments. By investigating developmental toxicity in embryos and larvae, physiological and biochemical responses, and changes in key regulatory pathways under exposure to different concentrations of TpPa, we aim to elucidate the toxic effects of TpPa and the underlying mechanisms of the toxicity. The findings will enrich the ecotoxicological database for COFs by contributing data regarding impacts on farmed and wild fish species, providing methodological references for subsequent assessments of TpPa and other novel COF materials for aquaculture water treatment applications. These outcomes will ultimately offer a scientific basis for the formulation of environmental safety standards for COF-based water treatment materials in fishery waters, thereby balancing water treatment efficiency with the healthy development of aquaculture organisms.

## 2. Materials and Methods

### 2.1. Preparation and Characterization of TpPa

N,N-dimethylformamide (purity: 99.5%) and *p*-phenylenediamine (purity: 99%) were purchased from Shanghai Macklin Biochemical Co., Ltd. (Shanghai, China). *P*-toluenesulfonic acid (purity: AR) was obtained from Shanghai Aladdin Biochemical Technology Co., Ltd. (Shanghai, China), and 1,3,5-triformylphloroglucinol (purity: 98.92%) was supplied by Shanghai Haohong Biomedical Technology Co., Ltd. (Shanghai, China).

TpPa was synthesized according to a previously reported method [34]. Briefly, 0.0486 g of *p*-phenylenediamine, 0.475 g of *p*-toluenesulfonic acid, and 0.063 g of 1,3,5-triformylphloroglucinol were accurately weighed. Then, *p*-phenylenediamine and *p*-toluenesulfonic acid were placed in a dry agate mortar and ground uniformly for 5 min to yield a white powder. Subsequently, 1,3,5-triformylphloroglucinol was added and ground with the mixture for another 10 min, producing a yellow powder. Then, 100 μL of distilled water was added, and grinding was continued for 5 min. The resulting solid was transferred to a glass Petri dish and rapidly heated in an oven at 170 °C for 5 min, after which the dish was removed and cooled to room temperature. The cooled solid was transferred to a centrifuge tube and washed alternately with N,N-dimethylformamide and distilled water 3–5 times, followed by centrifugation at 3500 rpm for 3–5 min, and the supernatant was discarded. The precipitate was then dried to constant weight in an oven at 60 °C to obtain the final product TpPa.

Then, X-ray diffraction (XRD) analysis was performed using an X-ray diffractometer (D8 Venture, Bruker, Bremen, Germany) over a scanning range of 2–40° (2θ) at a scan rate of 1°/min to identify the crystalline phase of TpPa. The morphological characteristics of the particles were observed using a scanning electron microscope (SEM, SU5000, Hitachi, Tokyo, Japan) and a transmission electron microscope (TEM, JEM-F200, JEOL, Akishima, Japan).

### 2.2. Zebrafish Culture, Embryo Collection, and Embryo Exposure

Wild-type adult zebrafish (TUB line) were obtained from Shanghai Feixi Biotechnology Co., Ltd. (Shanghai, China) and maintained in a thermostatic light incubator under the conditions listed in Table S1. To obtain embryos, male and female fish were paired at a ratio of 1:1 or 1:2 in a breeding tank equipped with a spawning insert the evening prior to spawning. Spawning was triggered by light onset the following morning. Fertilized eggs were collected within 1 h post-fertilization (hpf) and rinsed with E3 medium. Embryos were examined under a stereomicroscope, and only normally developed embryos at the blastula stage (2–4 hpf) with intact chorions and no signs of coagulation or developmental defects were selected for subsequent experiments.

A stock solution of TpPa (1 g/L) was prepared with ultrapure water, sonicated for 30 min to ensure homogeneity, and then serially diluted to exposure solutions at nominal concentrations of 0, 1, 5, 10, 20, and 50 mg/L. All solutions were freshly prepared before use. Selected embryos were randomly transferred to Petri dishes containing TpPa solutions at different concentrations. For each concentration, three replicate groups were established, with 100 embryos per group in disposable Petri dishes. All embryos were incubated at 28 °C in a thermostatic light incubator. Dead embryos and larvae were removed promptly during exposure to avoid secondary mortality, and exposure was terminated at 120 hpf.

### 2.3. Developmental Toxicity in Zebrafish Embryos and Larvae

The developmental toxicity endpoints were assessed according to OECD Test Guideline 236 (Fish Embryo Acute Toxicity Test) and the extended General Morphology Score system described in the zebrafish embryo developmental toxicity assay (ZEDTA) [35,36]. Embryo and larva mortality rates were observed and recorded at 24, 48, 72, and 96 hpf. Hatching status was examined at 48, 72, 96, and 120 hpf. Malformation types were recorded at 72, 96, and 120 hpf, and the number of malformed larvae was counted at 72 and 96 hpf. The three indicators above were all calculated based on the initial 100 embryos exposed in each group. For behavioral and morphological observations, 15 larvae per group were randomly selected at each time point. Spontaneous movement frequency was recorded within 1 min under a stereomicroscope (SW350T, Motic, Xiamen, China) at 24 hpf. At 48 and 72 hpf, the larvae were transferred to clean medium and allowed to stabilize. Heart rate was determined by counting the number of heartbeats within a 30 s period under a microscope. At 96 and 120 hpf, ventral images of larvae were captured under a stereomicroscope, and body length was measured.

### 2.4. Determination of Antioxidant Biomarkers in Zebrafish Larvae

After 120 h of exposure, fifty zebrafish larvae were collected for antioxidant index analysis, rinsed alternately with PBS and ultrapure water, blotted dry, weighed, and stored at −80 °C. For biochemical assays, the frozen larvae were homogenized in ice-cold PBS (1:9, *w*/*v*) and centrifuged at 10,000 rpm for 10 min at 4 °C. The supernatants were collected for subsequent analyses. The activities of catalase (CAT, Cat. No. A001-1-2) and superoxide dismutase (SOD, Cat. No. A007-1-1), as well as the content of malondialdehyde (MDA, Cat. No. A003-1-2), were determined using commercial assay kits purchased from Nanjing Jiancheng Bioengineering Institute (Nanjing, China).

### 2.5. Measurement of ROS Accumulation and Apoptosis in Zebrafish Larvae

At 72 hpf, 15 zebrafish larvae were randomly selected from each group to perform ROS detection and acridine orange (AO) staining. ROS accumulation and cell apoptosis were qualitatively and quantitatively analyzed using an ROS detection kit (Nanjing Jiancheng, Cat. No. E004-1-1) and AO staining. Larvae were processed according to the kit instructions and observed under a fluorescence microscope (Axio Observer, Zeiss, Oberkochen, Germany). Fluorescence images were photographed, and ImageJ software (v1.8.0.112) was used for quantitative analysis of fluorescence intensity to evaluate ROS levels and apoptotic cell death.

### 2.6. Gene Expression Analysis in Zebrafish Larvae

The remaining larvae were used for gene expression analysis, washed with RNase-free water, dried with absorbent paper, and stored at −80 °C until use. The expression levels of genes related to embryonic development, locomotor activity, antioxidant defense, and apoptosis were analyzed according to a previous study using a Bio-Rad CFX96 real-time quantitative PCR system (Bio-Rad, Hercules, CA, USA) [13]. The *β-actin* gene was used as an internal reference. Primer sequences and gene details were listed in Table S2.

Total RNA was extracted from frozen samples using Trizol reagent following the manufacturer’s protocol. RNA purity was verified using a NanoDrop 2000 UV-Vis spectrophotometer (Thermo, Waltham, MA, USA), with an OD_260_/OD_280_ ratio ≥ 1.8 indicating high purity. RNA was reverse-transcribed into cDNA using a Takara reverse transcription kit (RR037A, Kusatsu, Japan). Quantitative PCR amplification was performed under the following conditions: 1 cycle of pre-denaturation at 95 °C for 30 s; 40 cycles of denaturation at 95 °C for 10 s, annealing at 60 °C for 30 s, and extension and data collection at 72 °C for 30 s. Relative gene expression was calculated using the 2^−ΔΔCT^ method.

### 2.7. Data Analysis

Statistical analysis was performed using SPSS 22.0. Graphs were plotted using Origin 2024 and GraphPad Prism 8.0. For normally distributed data with homogeneity of variance, one-way analysis of variance (one-way ANOVA) followed by the least significant difference (LSD) test was used to determine significant differences among groups. Otherwise, nonparametric tests were applied. Asterisks indicate levels of significance (Table S3).

## 3. Results and Discussion

### 3.1. Characterization of TpPa

**Figure 1:**
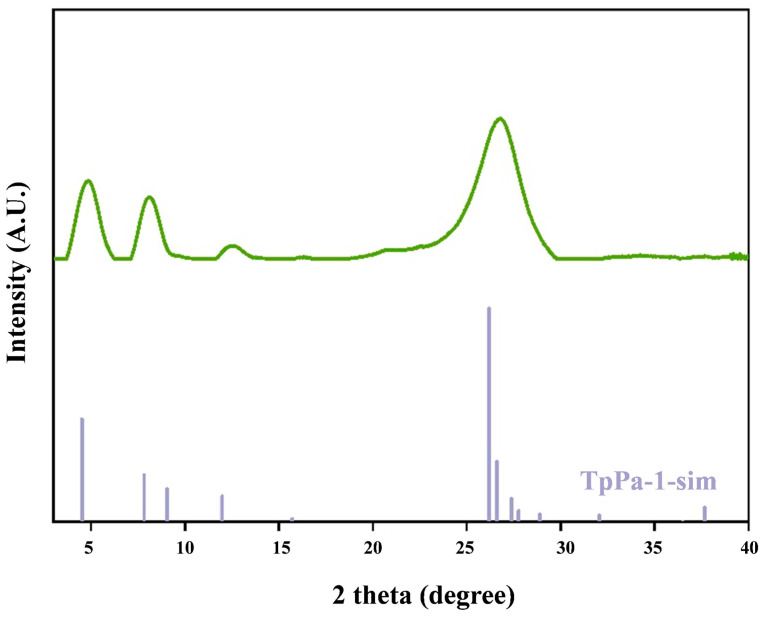
XRD pattern of TpPa.

Figure 1 shows the XRD pattern of the synthesized TpPa. Three strong diffraction peaks at 2θ = 4.82°, 8.10°, and 26.80° correspond to the (100), (200), and (001) crystal planes of the COF structure, respectively, which are consistent with the simulated XRD pattern of TpPa (TpPa-1-sim) [37].

**Figure 2:**
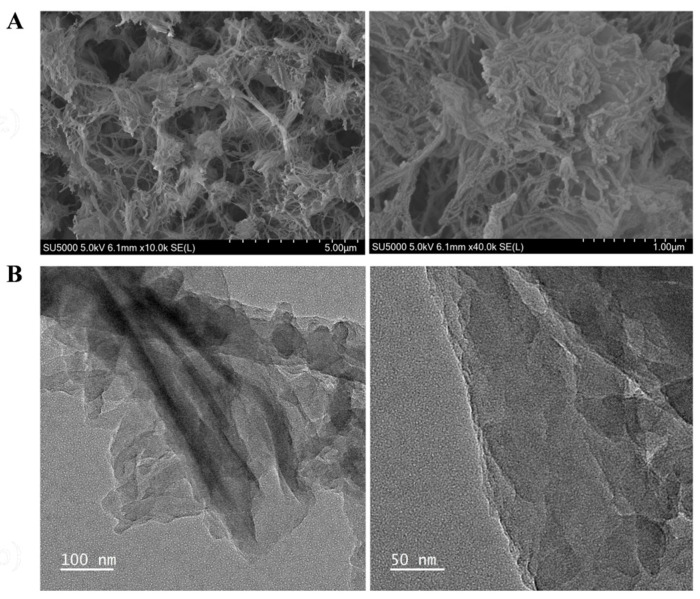
Characterization of TpPa. (**A**) SEM image of TpPa; (**B**) TEM image of TpPa.

The morphology and microstructure of TpPa were characterized by SEM and TEM. At low magnification (Figure 2A(left), ×10,000), the SEM image reveals a sponge-like porous architecture with abundant macropores ranging from 0.5 to 2 μm in diameter. At higher magnification (Figure 2A(right), ×40,000), the porous framework is shown to comprise intertwined nanofibers with diameters of approximately 20–50 nm, forming a three-dimensional network. TEM analysis further elucidated the intrinsic structure of TpPa. At lower magnification (Figure 2B(left), scale bar: 100 nm), the TEM image captured flexible nanosheets with thicknesses of 20–50 nm, which were wrinkled, folded, and partially overlapped, suggesting that the nanofibrous morphology observed by SEM arose from the curling and entanglement of these nanosheets. At higher magnification (Figure 2B(right), scale bar: 50 nm), the nanosheets exhibited a relatively smooth surface with visible wrinkles and bending edges, indicative of a layered, sheet-like structure. Overall, the material exhibits a typical porous framework feature of COFs [38,39].

### 3.2. Developmental Toxicity of TpPa to Zebrafish Embryos and Larvae

**Figure 3:**
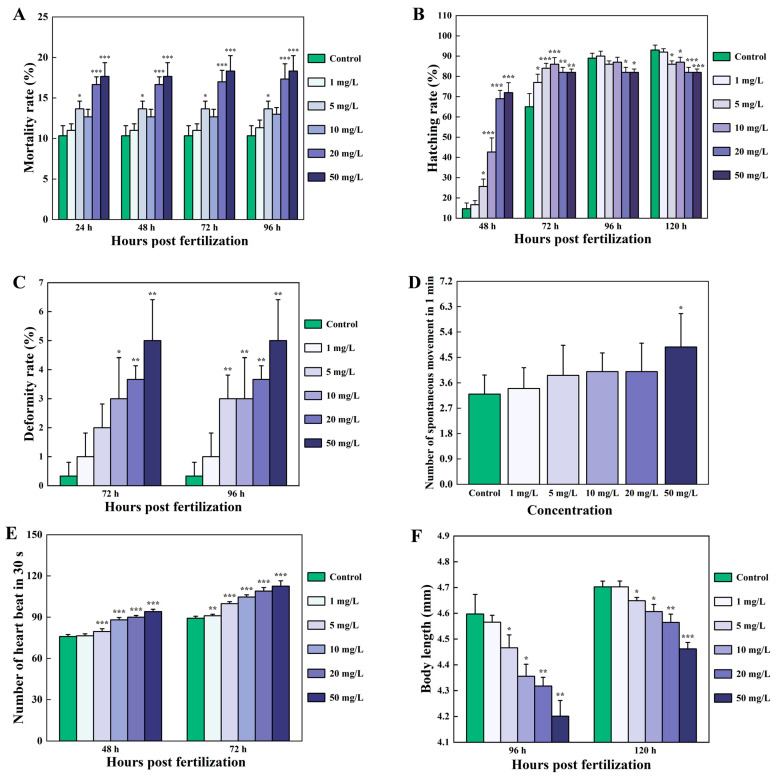
Developmental toxicity of TpPa in zebrafish embryos and larvae. (**A**) Mortality rate; (**B**) Hatching rate; (**C**) Deformity rate; (**D**) Spontaneous movement; (**E**) Heart rate; (**F**) Body length. Data are presented as mean ± SD. * *p* < 0.05, ** *p* < 0.01, *** *p* < 0.001 vs. control group.

TpPa exposure exerted significant concentration- and time-dependent effects on behavioral and survival-related endpoints in zebrafish embryos and larvae (Figure 3). As the concentration and exposure time changed, indicators such as the malformation rate, spontaneous movement frequency, and heart rate all changed accordingly. This was associated with the accumulation of nanoscale TpPa particles on the chorion surface and their penetration into embryonic tissues, causing continuous cellular damage [40].

During the 96 h exposure of zebrafish embryos to TpPa, mortality was observed exclusively at 24 hpf in all groups, with no further mortality occurring thereafter (Figure 3A). Compared with the control group, the 5 mg/L TpPa exposure group exhibited a significant increase in mortality rate (*p* < 0.05), while the 20 and 50 mg/L TpPa exposure groups showed highly significant increases (*p* < 0.001). As a nanoscale COF material, TpPa particles may adhere to the surface of the embryonic chorion through adsorption, thereby obstructing chorionic pore canals and causing hypoxia [23]. This effect manifests as an acute toxic response in embryos at 24 hpf.

Notably, at 48 hpf, the hatching rate increased significantly with rising TpPa concentration in all groups except the control and 1 mg/L groups, with the highest concentration exposure group exhibiting the maximum hatching rate (Figure 3B). This suggests that TpPa may induce a biphasic stress response during early embryonic development. On one hand, acute toxicity causes direct embryonic mortality. On the other hand, stress triggers premature chorion rupture. Normal hatching of zebrafish embryos depends on the proper secretion of hatching enzymes and degradation of the chorion [41]. It is speculated that TpPa may induce an embryonic stress response, promoting premature secretion of hatching enzymes, which in turn leads to early degradation of the chorion and premature hatching [42]. Meanwhile, although control group embryos initiated hatching later, their hatching enzyme system functioned normally with low mortality, enabling sustained hatching until 120 hpf. Consequently, the cumulative hatching number in the control group gradually surpassed and became significantly higher than that of all exposure groups after 72 hpf. Therefore, despite the transient increase in hatching rate observed in high-concentration groups at 48 hpf, the control group exhibited a significantly higher total hatching number than all exposure groups at 120 hpf, with the final hatching rate showing a concentration-dependent decrease.

**Figure 4:**
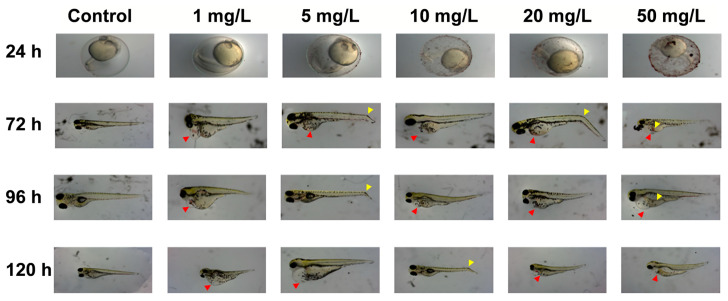
Morphology of normal and malformed larvae. Red arrows indicate pericardial edema, and yellow arrows indicate somite malformations.

Upon TpPa exposure, the main malformations included pericardial edema, spinal curvature, spinal tail malformation, and yolk sac enlargement, as shown by the arrows in Figure 3C and Figure 4. At 24 h post-exposure, due to the adsorptive property of the material, a substantial amount of TpPa powder adhered to the outer membrane of zebrafish embryos. This may have led to the retention of metabolic waste from the embryos, and the accumulation of toxic metabolites was most directly reflected as tissue edema. At 72 h, 96 h, and 120 h, numerous malformed individuals exhibited pericardial edema and yolk cyst formation, suggesting that the development of these malformations may be associated with the attachment of TpPa particles to the embryonic chorion prior to hatching.

Compared with the control group, the spontaneous movement frequency in the 50 mg/L exposure group was significantly increased (Figure 3D, *p* < 0.05), indicating that TpPa induced hyperactivity in the early motor nervous system development of zebrafish larvae. The spontaneous movement of zebrafish embryos around 24 h is the earliest motor behavior, driven by rhythmic electrical activity mediated by gap junctions of primary motor neurons [43]. Changes in movement frequency during this period directly reflect the developmental status and functional integrity of the spinal motor neuron circuit and have been established as a sensitive and reliable behavioral endpoint for assessing early neurodevelopmental toxicity [44]. In this study, the significant increase in larval spontaneous movement frequency following TpPa exposure is consistent with the findings of Pedersen et al., who reported hyperactive spontaneous movement in zebrafish exposed to polystyrene nanoplastics [45]. It is speculated that TpPa may induce mild stimulation of embryonic neural cells, triggering a compensatory response characterized by increased neuronal excitability [46]. Notably, the highest concentration of TpPa (50 mg/L) also resulted in the highest subsequent mortality rate, suggesting that this neuronal hyperexcitability is not a sign of normal development but rather an initial compensatory response of embryos to toxic stress. Prolonged exposure may exacerbate neural damage, ultimately leading to embryonic death.

As shown in Figure 3E, at 48 h post-exposure, the heart rate in the 1 mg/L group showed no significant difference from the control group (*p* > 0.05), while the 5–50 mg/L groups exhibited a dose-dependent increase. At 72 h, the increase in heart rate was further exacerbated, with heart rates in the 1 mg/L and higher exposure groups being significantly elevated compared with the control group. As the core functional organ during early zebrafish development, the heart initiates contraction at 26 h, completes ventricular differentiation by 48 h, and forms a complete vascular tree structure by 72 h [47]. Exposure to TpPa induced a significant increase in heart rate in zebrafish embryos, leading to excessive myocardial contraction. Excessive myocardial contraction dramatically increases oxygen consumption in cardiomyocytes, thereby triggering oxidative stress, which in turn further damages cardiomyocytes, forming a vicious cycle [48].

Body length was significantly reduced in the 5–50 mg/L groups at 96 hpf (*p* < 0.001) and remained inhibited at 120 hpf with no sign of recovery (Figure 3F). Previous studies have demonstrated that TpBD-Me_2_, a COF material structurally analogous to TpPa, exerts toxicity to zebrafish embryos primarily through particle deposition and physical damage rather than through specific chemical properties, even at extremely low concentrations [23]. TpPa may interfere with embryonic development through similar physicochemical mechanisms, including particle accumulation, membrane penetration, and physical injury. These findings suggest that TpPa residues in aquaculture water above 1 mg/L could compromise fish development, with concentrations of 5 mg/L and above causing significant increases in mortality, malformation rate, and growth inhibition, particularly during the critical hatchery stage when embryos are most vulnerable to particle adsorption and physical damage.

### 3.3. Effects of TpPa on ROS Levels, Antioxidant Indices, and Apoptosis in Zebrafish Larvae

**Figure 5:**
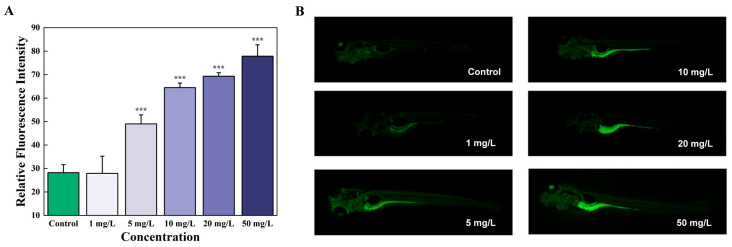
Effects of TpPa on ROS levels in zebrafish larvae. (**A**) Analysis of relative ROS fluorescence intensity; (**B**) Representative fluorescence images of ROS staining in zebrafish larvae under different TpPa concentrations. Data are presented as mean ± SD. *** *p* < 0.001 vs. control group.

Mitochondria generate ATP through oxidative phosphorylation, but during this process, electrons leaking from the electron transport chain can react with oxygen to produce superoxide anions and other ROS [49]. Excessive accumulation of ROS can trigger the mitochondria-mediated apoptotic pathway. This pathway involves initial depolarization of the mitochondrial membrane potential and cytochrome c release, subsequent activation of apoptotic execution molecules such as *Caspase9* and *Caspase3*, and ultimate induction of programmed cell death [50]. In addition, ROS can promote apoptosis through activation of the *p53* tumor suppressor and modulation of the *Bcl2*/*Bax* ratio [51]. Therefore, TpPa-induced ROS overaccumulation may represent the core mechanism underlying embryonic cell apoptosis and developmental toxicity. As shown in Figure 5, fluorescence intensity in zebrafish larvae was significantly increased by 1.73-, 2.28-, 2.45-, and 2.76-fold after 72 hpf exposure to 5, 10, 20, and 50 mg/L TpPa, respectively (*p* < 0.001). ROS signals were mainly distributed in the abdominal region, consistent with previous reports that nanomaterial-induced ROS accumulates in the lipid-rich, metabolically active yolk sac [52]. TpPa may accumulate in this area and disturb mitochondrial function, leading to excessive ROS production and subsequent yolk sac edema. Furthermore, the accumulation of ROS was consistent with the increased heart rate and the occurrence of pericardial edema observed in the above results [53,54,55]. It is speculated that TpPa may induce stress in cardiomyocytes, leading to an increased contraction frequency to meet the oxygen metabolism demand under toxic stress.

**Figure 6:**
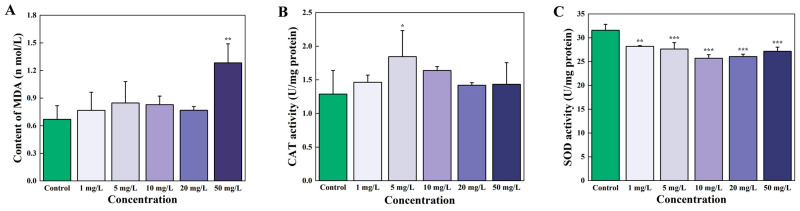
Effects of TpPa on antioxidant indices of zebrafish larvae. (**A**) MDA content (n mol/L); (**B**) CAT activity (U/mg protein); (**C**) SOD activity (U/mg protein). Data are presented as mean ± SD. * *p* < 0.05, ** *p* < 0.01, *** *p* < 0.001 vs. control group.

When organisms are subjected to oxidative stress, ROS attack unsaturated fatty acids in biological membranes, initiating lipid peroxidation chain reactions that ultimately generate MDA [56,57]. MDA can cross-link with biomacromolecules such as proteins and nucleic acids, disrupting their structure and function [57]. Therefore, elevated MDA levels indicate more severe oxidative damage, and the degree of lipid peroxidation serves as a critical biomarker for assessing oxidative injury [58]. As shown in Figure 6, MDA content remained unchanged in zebrafish larvae exposed to 1–20 mg/L TpPa but increased significantly at 50 mg/L (*p* < 0.01), indicating severe oxidative damage under high-concentration exposure.

CAT primarily catalyzes the decomposition of hydrogen peroxide (H_2_O_2_) into water (H_2_O) and oxygen (O_2_) [59]. Classical theory posits that CAT and SOD constitute the first enzymatic antioxidant defense line in cells, with SOD scavenging superoxide anions and CAT eliminating the resulting H_2_O_2_ [60]. However, the present study revealed divergent activity trends. SOD activity decreased significantly across all tested concentrations, whereas CAT activity increased only at 5 mg/L and subsequently declined to control levels at higher concentrations. This discrepancy indicates that the SOD-CAT antioxidant axis does not operate as a strictly coupled system under TpPa-induced oxidative stress.

This uncoupling can be attributed to the multiple sources of H_2_O_2_ and the distinct subcellular localizations of these enzymes. Although H_2_O_2_ is the product of SOD-catalyzed reactions, it can also be generated by various SOD-independent cellular sources, including the mitochondrial electron transport chain, NADPH oxidases, and peroxisomes [61,62,63]. These alternative sources continue to drive intracellular H_2_O_2_ accumulation even when SOD is suppressed, thereby activating CAT. Furthermore, CAT is predominantly localized in peroxisomes, whereas SOD is distributed in mitochondria and the cytosol [64]. This spatial segregation enables CAT to respond to local H_2_O_2_ accumulation within peroxisomes independently of SOD-derived H_2_O_2_ from mitochondria or the cytosol.

This spatial and functional independence manifests as distinct concentration-dependent stages. At 1 mg/L, SOD was suppressed without CAT activation or elevated MDA levels, suggesting that TpPa exerts a high-affinity toxic effect on SOD as the core mechanism of TpPa-induced oxidative stress. At 5 mg/L, SOD remained suppressed, but CAT was significantly activated, successfully clearing accumulated H_2_O_2_ and preventing oxidative damage. At 10–20 mg/L, CAT activity declined to control levels while SOD remained suppressed, yet MDA did not increase significantly. The decline in CAT activity at high concentrations may be attributed to direct enzyme damage caused by elevated H_2_O_2_ levels or compensatory upregulation of alternative enzymes [65,66,67]. At 50 mg/L, MDA accumulated significantly, indicating complete collapse of the antioxidant defense system and uncontrolled oxidative damage.

**Figure 7:**
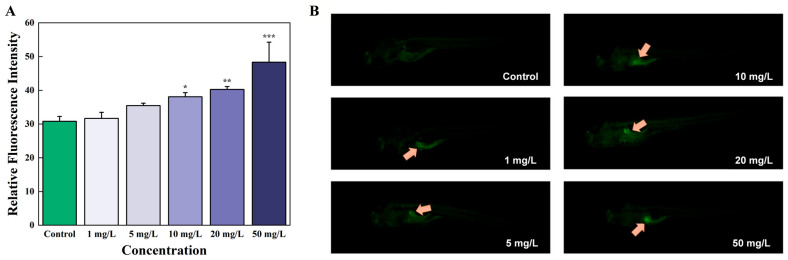
Effects of TpPa exposure on apoptosis in zebrafish larvae. (**A**) Analysis of relative AO staining fluorescence intensity; (**B**) Representative fluorescence images of AO staining in zebrafish larvae under different TpPa concentrations, and arrows indicate AO-positive apoptotic cells (bright green fluorescence). Data are presented as mean ± SD. * *p* < 0.05, ** *p* < 0.01, *** *p* < 0.001 vs. control group.

AO staining results (Figure 7) revealed elevated fluorescence intensity in all treatment groups, in good agreement with ROS accumulation and mortality patterns, suggesting that TpPa triggers apoptosis via oxidative stress. Normal larvae showed uniform green fluorescence, while apoptotic cells exhibited bright green fluorescence indicative of nuclear condensation. Obvious apoptosis was observed from 10 mg/L onward, primarily in the yolk sac and heart, corresponding well with pericardial edema and elevated heart rate. At low concentrations (1 and 5 mg/L), ROS increased without marked apoptosis, indicating early cellular damage below the apoptotic threshold, whereas high concentrations overwhelmed defense systems and induced severe apoptosis.

### 3.4. Effects of TpPa on the Expression of Key Functional Genes

**Figure 8:**
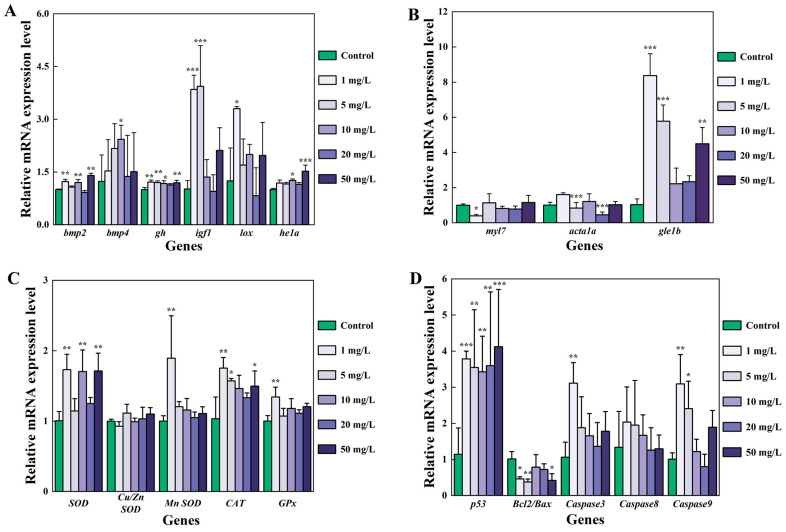
Effects of TpPa on the expression of key functional genes related to zebrafish development. (**A**) Growth and development-related genes; (**B**) Locomotion-related genes; (**C**) Antioxidant-related genes; (**D**) Apoptosis-related genes. Data are presented as mean ± SD. * *p* < 0.05, ** *p* < 0.01, *** *p* < 0.001 vs. control group.

To reveal the toxic mechanism of TpPa on zebrafish larvae, expression levels of genes related to growth and development, locomotion, antioxidant activity, and apoptosis were detected, with results shown in Figure 8.

For growth and development-related genes, the upregulation of *bmp2* and *bmp4* (*p* < 0.05) indicated abnormal skeletal development in zebrafish [68]. In the low-concentration (1 and 5 mg/L) groups, compensatory upregulation of *gh* and *igf1* was observed to alleviate TpPa-induced growth inhibition [69]. Notably, *igf1* expression in the 1 and 5 mg/L groups increased significantly (*p* < 0.001), reaching approximately 3.9–4.0 times that of the control group. However, at higher concentrations (20–50 mg/L), *igf1* levels returned to baseline, accompanied by significant body length suppression (*p* < 0.01). This paradoxical pattern suggests that the compensatory capacity of the *GH*/*IGF* axis was overwhelmed at high TpPa concentrations, likely due to receptor desensitization or downstream signaling blockade, resulting in the failure of growth-promoting effects despite normalized *igf1* levels and ultimately leading to irreversible growth retardation [70]. Upregulation of *lox* was observed exclusively at 1 mg/L (*p* < 0.05), whereas no significant changes were observed at 5–50 mg/L. However, the malformation rate increased significantly at concentrations ≥ 5 mg/L (*p* < 0.01). This dissociation between *lox* expression and malformation incidence suggests that the compensatory upregulation of *lox* is confined to a narrow concentration window and fails to respond at higher doses. The high expression of *he1a* may be attributed to disruption of the normal transcriptional regulatory negative feedback loop, causing *he1a* expression to escape the post-hatching shutdown control [71]. Premature hatching consequently leads to incomplete organ development in larvae.

For locomotion-related genes, *myl7* was significantly inhibited under low-concentration (1 mg/L) TpPa exposure, indicating that myocardial function was impaired at this stage. The slight increase in heart rate in the low-concentration group may be a compensatory response of the body to myocardial injury. The expression of *myl7* rebounded in the high-concentration groups, suggesting a compensatory mechanism to enhance myocardial contractility and maintain stable circulatory function. However, this compensatory effect failed to alleviate cardiac injury, ultimately leading to sustained tachycardia and aggravated pericardial edema. Unexpectedly, *acta1a* expression was significantly downregulated in the 5 and 20 mg/L groups (*p* < 0.05), whereas spontaneous movement frequency at 24 hpf remained comparable to that of the control. This dissociation between gene expression and functional phenotype is consistent with genetic compensation within the *actin* gene family, wherein downregulation of one isoform triggers compensatory upregulation of paralogues (e.g., *acta1b* or *actc1a*) to buffer muscle function [72]. Notably, at 50 mg/L, spontaneous movement frequency increased significantly (*p* < 0.05), likely reflecting neurotoxicity-induced hyperexcitability of motor circuits rather than enhanced muscle function. The expression of *gle1b* showed an extremely significant upward trend in all TpPa exposure groups, peaking at approximately 8.4-fold of the control in the 1 mg/L group (*p* < 0.001), followed by the 5 mg/L group (about 5.7-fold, *p* < 0.001), and remaining significantly elevated at 4.5-fold of the control in the 50 mg/L group (*p* < 0.01). Sustained high expression of *gle1b* indicates that TpPa strongly interferes with angiogenesis and neuromotor regulatory pathways. This is closely related to the previously observed abnormal behavioral phenotypes, such as significantly increased spontaneous movement frequency and tachycardia, and may be one of the core molecular mechanisms underlying TpPa-induced early embryonic hyperactivity.

For antioxidant-related genes, the expression of *SOD*, *Cu/Zn SOD*, and *Mn SOD* all showed a significant upward trend in all TpPa exposure groups compared with the control group. *SOD* expression was extremely significantly elevated in the 1, 10, and 50 mg/L groups (*p* < 0.01), reaching 1.7–1.8-fold that of the control group. No significant differences were observed in *Cu/Zn SOD* across all concentration groups. Compared with the control group, *Mn SOD* expression in the 1 mg/L group showed a significant increase to 1.8-fold (*p* < 0.01). Partial upregulation of the *SOD* family genes represents a transcriptional compensatory response of the body to oxidative stress, suggesting an attempt to enhance enzyme activity and eliminate excessive ROS by increasing gene expression [73]. However, the sustained inhibition of SOD enzyme activity indicates that TpPa may inhibit the translation process of *SOD* or disrupt its spatial structure, preventing transcriptional activation from translating into effective enzyme activity. This decoupling between transcription and activity has been reported under various environmental stresses. For example, although *SOD* gene expression was significantly upregulated under phthalate exposure, enzyme activity decreased, suggesting that molecular binding interactions or translational inhibition mechanisms may be involved [74]. Similar findings have been reported under salt stress, demonstrating that antioxidant enzyme activities were inhibited despite the upregulation of their encoding genes, potentially due to membrane structural damage and metabolic interference induced by stress [75].

In the 1–50 mg/L TpPa exposure groups, *CAT* expression was upregulated compared with the control group. The expression of *CAT* was extremely significantly elevated in the 1 mg/L group (*p* < 0.01), reaching approximately 1.7-fold that of the control, and remained significantly higher in the 5 and 50 mg/L groups (*p* < 0.05), at approximately 1.5-fold the control level. Notably, CAT enzyme activity was also significantly increased in the 5 mg/L group, reflecting compensatory activation of the antioxidant system. However, in the high-concentration group, enzyme activity returned to the control level, whereas gene expression remained upregulated, suggesting that transcriptional activation of *CAT* at high concentrations failed to translate into effective enzymatic activity, thereby being unable to counteract oxidative damage induced by hydrogen peroxide accumulation. In the 1–50 mg/L TpPa exposure groups, *GPx* expression was consistently higher than that of the control group, with extremely significant upregulation observed in the 1 mg/L group (*p* < 0.01), reaching 1.4-fold the control level, while the remaining concentration groups exhibited mild upregulation. MDA content showed no significant change in the low- to medium-concentration groups, whereas *GPx* expression was upregulated, indicating that larvae activated the *GPx* pathway to suppress lipid peroxidation. In the high-concentration group, MDA content was significantly elevated. Despite sustained upregulation of *GPx* expression, this was insufficient to offset lipid peroxidation damage triggered by excessive ROS, ultimately leading to aggravated oxidative stress.

For apoptosis-related genes, *p53* expression showed an extremely significant upward trend in all TpPa exposure groups compared with the control, significantly increasing to 3.7-fold of the control in the 1 mg/L group (*p* < 0.001) and peaking at 4.1-fold of the control in the 50 mg/L group (*p* < 0.001) with increasing concentration. This is consistent with the aforementioned ROS accumulation results, indicating that TpPa induces DNA damage by triggering oxidative stress, thereby activating the *p53* pathway and initiating the apoptotic program. The *Bcl2*/*Bax* ratio showed a significant decline in all exposure groups, indicating that TpPa triggers cell apoptosis during zebrafish embryonic development by inhibiting anti-apoptotic signals and enhancing pro-apoptotic signals. This further confirms that *Bcl2*/*Bax* pathway imbalance is a key factor in TpPa-induced apoptosis. Compared with the control group, *Caspase3* expression peaked at 3.1-fold of the control in the 1 mg/L group (*p* < 0.01). *Caspase8* expression rose to 2.1-fold of the control group in the 20 mg/L group but was not statistically significant (*p* = 0.678). *Caspase9* expression was extremely significantly increased in the 1 mg/L group (*p* < 0.01), approximately 3.2-fold of the control group. These results indicate that TpPa simultaneously activates both the death receptor and mitochondrial apoptotic pathways, with downstream *Caspase3* executing apoptosis. This is highly consistent with the phenotype of apoptotic cells concentrated in the yolk sac and heart regions observed in AO staining, directly corresponding to malformation characteristics such as embryonic pericardial edema and yolk sac enlargement.

The molecular evidence confirms that TpPa mediates developmental toxicity in zebrafish through synergistic multi-pathway effects. TpPa abnormally activates the *bmp2*/*bmp4* and *GH*/*IGF* growth regulatory pathways, causing skeletal differentiation disorder and growth arrest in zebrafish. It induces early embryonic hyperactivity and abnormal heart rate by continuously upregulating the expression of neuromotor regulatory genes such as *gle1b*. At the transcriptional level, it triggers compensatory upregulation of antioxidant genes such as *SOD* and *CAT*, but inhibits their antioxidant function at the translational and enzymatic activity levels, exacerbating oxidative stress injury. Meanwhile, it activates the *p53*-mediated stress pathway, downregulates the *Bcl2*/*Bax* ratio, breaks the apoptotic balance, and ultimately induces apoptosis and developmental malformations in zebrafish larvae.

Collectively, these molecular findings elucidate the multi-pathway mechanisms underlying TpPa-induced developmental toxicity. It should be noted that the environmental concentrations of TpPa remain unknown due to the lack of monitoring data, and the tested concentrations were selected to establish dose–response relationships rather than to simulate specific environmental scenarios. Nevertheless, these findings provide critical baseline data for future risk assessment as COF production and application expand.

## 4. Conclusions

As the first aquatic toxicity assessment of TpPa, this study found that TpPa induced concentration- and time-dependent zebrafish toxicity through adsorption and penetration, progressing via ROS overaccumulation, antioxidant collapse, and apoptotic activation. Notably, the decoupling of transcriptional activation from enzymatic inhibition in SOD and CAT systems revealed that TpPa may disrupt cellular defense by interfering with post-translational modification or protein conformation. The biphasic response of the *GH*/*IGF* axis, featuring compensatory activation at low concentrations but desensitization that mediated failure at high concentrations, elucidated the critical mechanism of transition from compensation to decompensation in developmental toxicity. These findings provide critical data for assessing the environmental safety and ecological risks of TpPa, with implications for the monitoring and regulation of COF contamination in aquaculture water systems, thereby safeguarding farmed aquatic animals from exposure-related toxicity and ensuring production sustainability.

## Data Availability

Data are available upon request.

## Supplementary Materials

The following supporting information can be downloaded at: https://www.mdpi.com/article/10.3390/ani16152282/s1, Table S1. Zebrafish husbandry conditions and embryo medium composition; Table S2. Primer sequences of functional genes used for zebrafish larvae; Table S3. Significance levels of statistical results.
