# Supplementary material for: Toxicity of the Covalent Organic Framework Material TpPa to Zebrafish
Source: Animals (Basel). 2026 Jul 23;16(15):2282. doi: 10.3390/ani16152282 (PMC13463574; doi:10.3390/ani16152282)
Supplement: Supplementary file 1 [file animals-16-02282-s001.zip › animals-4389324-supplementary.pdf]

Supporting Information

Toxicity of the Covalent Organic Framework Material TpPa to Zebrafish

Cuiming Huang [1,†], Jingui Lin [1,†], Xiudan Yang [1], Lu Gan [2], Lijie Xu [3], Muting Yan [1,*] and Han Gong [1,4,*]

[1] Joint Laboratory of Guangdong Province and Hong Kong Region on Marine Bioresource Conservation and Exploitation, South China Agricultural University, Guangzhou 510642, China; huangcuiming0124@163.com (C.H.); jingui_lin@163.com (J.L.); yangxiudan0020@163.com (X.Y.)

[2] College of Materials Science and Engineering, Nanjing Forestry University, Nanjing 210037, China; ganlu@njfu.edu.cn

[3] College of Ecology and Environment, Nanjing Forestry University, Nanjing 210037, China; xulijie@njfu.edu.cn

[4] Department of Chemistry, Stockholm University, 10691 Stockholm, Sweden

* Correspondence: marineymt@scau.edu.cn (M.Y.); han.gong@connect.polyu.hk (H.G.)

† These authors contributed equally to this work.

Table S1. Zebrafish husbandry conditions and embryo medium composition

Environmental Factor	Condition
Temperature	28 ± 0.5 ℃
Daily Light Cycle	14 h light/10 h dark
Daily Brine Shrimp Feeding Time	9:30 a.m.; 18:30 p.m.
pH	6.5-7.5
E3 Medium	5 mM NaCl, 0.17 mM KCl, 0.33 mM $CaCl_2$, 0.33 mM $MgSO_4$, diluted to 1 L

Table S2. Primer sequences of functional genes used for zebrafish larvae

Target Gene	Primer Sequence (5’-3’)
β-actin	F: CGAGCAGGAGATGGGAACC<br>R: CAACGGAAACGCTCATTGC
Growth and Development-related Genes	
bmp2	F: CGGGTCATCGTTTTCAGAGT<br>R: TACAGTGATTCGGGGAGACA
bmp4	F: ACGCTTTCTTCTTCCCTTCC<br>R: CGTAACATTCGCTTGAGGAG
gh	F: CAGTAGGAGGTCCAGATTCGTG<br>R: AGCCCTGTCCAGTGATTGGTATG
igf1	F: CGGTTTCTCTTGTCTCTCTCAG<br>R: GGCAAATCTCCACGATCTCTAC
Lox	F: ACGACATGCGTATGTTGCTGAGGTT<br>R: CTCTTCTGTGAGTGGTTGCGTCCAG
he1a	F: AACTGGTGGCAAACAGGTGGTCTC<br>R: TCGCTCCTGGATTGCTCATGGTAGA
Swimming and Locomotion-related Genes	
myl7	F: ATGGCACAGACCCAGAGG<br>R: GGGCAGCAGTTACAGACAGA
acta1a	F: ACGAGCAGAGGCAGAACC<br>R: ACCAGCCAAGTCCAAACG
gle1b	F: AGAAGCTGAGAGAGGAACAC<br>R: CTCCTGTTCTTTCTTCTTCTTC
Antioxidant Enzyme-related Genes	
SOD	F: GGCCAACCGATAGTGTTAGA<br>R: CCAGCGTTGCCAGTTTTTAG
CAT	F: AGACAAGATCACAGGCCAGAGCTT<br>R: TGGTCTGCAGTTTGAGACCGTTGA

Continued Table S2.

Target Gene	Primer Sequence (5'-3')
GPx	F: AGATGTCATTCCTGCACACG<br>R: AAGGAGAAGCTTCCTCAGCC
Cu/Zn SOD	F: GTCGTCTGGCTTGTGGAGTG<br>R: TGTCAGCGGGCTAGTGCTT
Mn SOD	F: CCGGACTATGTTAAGGCCATCT<br>R: ACACTCGGTTGCTCTCTTTTCTCT
Apoptosis-related Genes	
p53	F: GGGCAATCAGCGAGCAAA<br>R: ACTGACCTTCCTGAGTCTCCA
Bcl-2	F: TCACTCGTTCAGACCCTCAT<br>R: ACGCTTTCCACGCACAT
Bax	F: GGCTATTTCAACCAGGGTTCC<br>R: TGCGAATCACCAATGCTGT
Caspase3	F: ACTGGATCCTGGTGTGGAAACTGA<br>R: CCTGGTCATGATCTGCAAGAGC
Caspase8	F: GCTGATTTCCTGATCGGAATGGC<br>R: GTGTGTCCAGTCAATCCTTCAGC
Caspase9	F: GAAGACGGCGAAATCGATGC<br>R: CTGGCGGTTCTGACAACTTCC

Table S3. Significance levels of statistical results

Symbol	Meaning	*P*-value range
*	Significant difference	$P < 0.05$
**	Highly significant difference	$P < 0.01$
***	Extremely significant difference	$P < 0.001$
